# Current statement and safe implementation of minimally invasive surgery in the pancreas

**DOI:** 10.1002/ags3.12366

**Published:** 2020-07-09

**Authors:** Hiromitsu Hayashi, Hideo Baba

**Affiliations:** ^1^ Department of Gastroenterological Surgery Graduate School of Life Sciences Kumamoto University Kumamoto Japan

**Keywords:** distal pancreatectomy, learning curve, minimally invasive surgery, pancreas, pancreaticoduodenectomy

## Abstract

Minimally invasive pancreatic resection has become very popular in modern pancreatic surgery. Evidence of the benefits of a minimally invasive approach is accumulating thanks to prospective and randomized controlled studies. Minimally invasive surgery provides advantages to the surgeon due to the high definition of the surgical field and the freedom of fine movement of the robot, but should be considered only in selected patients and in high‐volume centers. Minimally invasive distal pancreatectomy for benign and low‐grade malignant tumors has established a secure position over open distal pancreatectomy, since it is associated with a shorter hospital stay, reduced blood loss, and equivalent complication rates. Minimally invasive distal pancreatectomy for pancreatic ductal adenocarcinoma appears to be a feasible, safe, and oncologically equivalent technique in experienced hands. On the other hand, the feasibility and safety of minimally invasive pancreaticoduodenectomy are still controversial compared with open pancreaticoduodenectomy. The choice of either technique among open, laparoscopic, and robotic approaches depends on surgeons' experience and hospital resources with a focus on patient safety. Further studies are needed to prove the perioperative and oncological advantages of minimally invasive surgery compared to open surgery in the pancreas. Here, we review the current status of minimally invasive pancreatic surgery and its safe implementation.

## Graphical abstract

Safe implementation of minimally invasive pancreatic surgery and its learning curve.

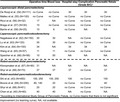

## 1 INTRODUCTION

Minimally invasive pancreatic resection (MIPR) has become very popular even in pancreatic surgery. Laparoscopic pancreatoduodenectomy (LPD) and laparoscopic distal pancreatectomy (LDP) were introduced in 1994 and 1996, respectively.^1^, ^2^ Robotic surgical systems had also already been established 20 years ago, and the first case of robotic distal pancreatectomy (RDP) was reported in 2002.^3^ Nowadays, laparoscopic and robotic approaches are becoming popular in MIPR, and the Miami international evidence‐based guidelines on MIPR were announced in 2020.^4^ In the guidelines, minimally invasive distal pancreatectomy (MIDP) for benign and low‐grade malignant tumors established a secure position over open distal pancreatectomy (ODP), since the former is associated with a shorter hospital stay, reduced blood loss, and equivalent complication rates. Both laparoscopic and robotic DP can be safe and feasible options. MIDP for pancreatic ductal adenocarcinoma (PDAC) appears to be a feasible, safe, and oncologically equivalent technique in experienced hands. On the other hand, the feasibility and safety of minimally invasive pancreaticoduodenectomy (MIPD) is still controversial compared with open pancreaticoduodenectomy (OPD). The choice of either technique among open, laparoscopic, and robotic approaches should be based on surgeons’ experience and hospital resources with a view to patient safety. Thanks to the accumulation of prospective and randomized controlled trials (RCTs), evidence of the benefits of MIPR is steadily increasing. Here, we review the current status of MIPR and its safe implementation.

## 2 MINIMALLY INVASIVE DISTAL PANCREATECTOMY (MIDP)

Distal pancreatectomy has traditionally been performed using an open approach. In the past decade, the minimally invasive approach using laparoscopic surgery or robot‐assisted surgery has become increasingly popular.^5^, ^6^ Pooled data of observational studies, generally from single, high‐volume expert centers, have suggested that MIDP is associated with a shorter length of hospital stay compared with ODP.^7^, ^8^ Despite this potential benefit, MIDP is only used in about one‐third of patients, according to a recent analysis of the National Surgical Quality Improvement Program (NSQIP) database.^9^ In Japan, a multicenter prospective registration study including 1197 LDPs revealed that postoperative morbidity and 90‐day mortality rates after LDP were 17% and 0.3%, respectively.^10^ Thus, LDPs are performed safely in Japan, especially in experienced institutions. The first multicenter, patient‐blinded, randomized controlled trial (LEOPARD study) demonstrated enhanced functional recovery after MIDP compared with ODP.^11^ MIDP also reduced operative blood loss, delayed gastric emptying, hospital stay, and adverse effect on postoperative quality of life.^11^ Additionally, MIDP (excluding the robotic approach) has been reported to be at least as cost‐effective as ODP.^12^ Cosmesis and quality of life were similar in MIDP and OPD 1 year after surgery.^12^

On the other hand, the clinical application of MIDP for PDAC is still controversial. Radical (R0) resection and enough lymph node retrieval are important for PDAC. The DIPLOMA trial “Minimally Invasive vs Open Distal Pancreatectomy for Ductal Adenocarcinoma: a Pan‐European Propensity Score Matched Study” investigated 1212 patients with DP from 34 centers in 11 countries.^13^ Its main outcomes were radical (R0) resection, lymph node retrieval, and survival. Of 356 (29%) MIDP patients, the conversion rate was 19% (65 of 356 cases), and the reasons were bleeding (26%), tumor advancement (23%), vascular involvement (26%), insufficient overview (6%), technical reason (5%), and unknown (12%). After matching (MIDP and ODP each with 340 cases) using age, sex, body mass index, American Society of Anesthesiologists physical status, previous abdominal surgery, neoadjuvant therapy, year of surgery, tumor size, involvement of other organs, and tumor location, MIDP showed less blood loss and shorter hospital stay compared to ODP, whereas Clavien–Dindo grade ≥3 complications and 90‐day mortality were comparable for MIDP and ODP. Although the R0 resection rate was significantly higher in MIDP, Gerota's fascia resection was less frequent and lymph node retrieval was lower after MIDP compared to ODP. Median survival time in a propensity score‐matched cohort was comparable for MIDP and ODP (29 and 31 months, respectively).^13^

To compare oncologic outcomes between MIDP (laparoscopic or robot‐assisted) and ODP in patients with PDAC, another systematic review screened 1760 studies and then included 21 studies with 11 246 patients.^14^ In this review, although overall survival, R0 resection rate, and use of adjuvant chemotherapy were comparable for MIDP and ODP, the retrieved lymph node was significantly lower in MIDP. Additionally, patients undergoing MIDP were more likely to have smaller tumors, less perineural invasion, and less lymphovascular invasion, reflecting earlier stage disease as a result of treatment allocation bias. The systematic review^14^ concluded that MIDP for PDAC was associated with comparable survival, R0 resection, and use of adjuvant chemotherapy, but a lower lymph node retrieval as compared to ODP. To overcome the issue of Gerota's fascia resection and lymph node retrieval during MIDP, the laparoscopic radical antegrade modular pancreatosplenectomy procedure for left side PDAC may be applicable.^15^ MIDP has spread internationally, and the oncological outcomes are mainly elucidated by prospective, observational series.^13^, ^16^, ^17^, ^18^, ^19^, ^20^ Due to the absence of RCTs, the oncologic efficacy of MIDP for PDAC remains unclear. In the current status, MIDP for PDAC provides equivalent results in terms of lymph node retrieval and positive margin status and comparable survival compared to ODP (Table 1). A further RCT to confirm the oncological safety of MIDP for PDAC is needed.

**TABLE 1:** Oncologic outcomes of minimally invasive pancreatic surgery for pancreatic ductal adenocarcinoma

Distal pancreatectomy						
Approach	Tumor size (mm)	Retrieved LN	R0 (%)	Adjuvant (%)	1‐y OS (%)	3‐y OS (%)
Shin et al^18^ 2015						
LDP (n = 70)	30 (0.4‐8.5)	12 (1‐34)	75.7	78.6	87.6	32.5 (5‐y OS)
ODP (n = 80)	35 (0.5‐14.0)	10 (1‐64)	83.8	68.8	74.0	27.6 (5‐y OS)
Stauffer et al^19^ 2016						
LDP (n = 44)	36 (0.5‐7.5)	25.9 (5‐48)	95.5	75.6	69	41
ODP (n = 28)	45 (0.2‐15)	12.7 (1‐45)	82.1	75	78	44
Sahakyan et al^16^ 2017						
LDP (n = 262)	30 (0.6‐9.0)	12 (1‐46)	87.1	68.9	NA	47
Raoof et al^20^ 2018						
MIDP (RDP) (n = 99)	35 (24‐45)	11 (5‐20)	84	60	70	46
MIDP (LDP) (n = 605)	37 (26‐50)	12 (6‐18)	85	59	80	43
Van Hilst et al^13^ 2019						
Total cohort						
MIDP (n = 356)	34 (25‐45)^a^	14 (8‐22)^a^	67	74	NA	NA
ODP (n = 856)	34 (23‐47)^a^	18 (11‐28)^a^	60	73	NA	NA
Propensity score matched cohort						
MIDP (n = 340)	35 (25‐45)^a^	14 (8‐22)^a^	67	76	29 mo (MST)	
ODP (n = 340)	30 (23‐45)^a^	22 (14‐31)^a^	58	73	31 mo (MST)	

Pancreaticoduodenectomy						
Approach	Tumor size (mm)	LN harvest	R0 (%)	Adjuvant (%)	1‐y OS (%)	3‐y OS (%)
Croome et al^32^ 2014						
LPD (n = 108)	33 ± 10	21.4 ± 8.1	77.8	76.0	25.3 mo (MST)	
OPD (n = 214)	33 ± 13	20.1 ± 7.5	76.6	76.0	21.8 mo (MST)	
Dokmak et al^33^ 2015						
LPD (n = 15)	24 (15‐40)	20 (8‐59)	60.0	NA	NA	NA
OPD (n = 14)	28 (25‐40)	25 (8‐47)	50.0	NA	NA	NA
Song et al^34^ 2015						
LPD (n = 11)	28 ± 6	15 ± 10	72.7	81.8	NA	53.6 (5‐y OS)
OPD (n = 261)	30 ± 12	16.2 ± 9.6	81.0	69.7	NA	28.8 (5‐y OS)
Delitto et al^35^ 2016						
LPD (n = 28)	NA	NA	NA	NA	20.7 mo (MST)	
OPD (n = 22)	NA	NA	NA	NA	21.1 mo (MST)	
Nussbaum et al^36^ 2016						
LPD (n = 1191)	33.7	17.4 (10.0)^b^	79.8	77.5	NA	NA
OPD (n = 6776)	33.6	16.5 (9.6)^b^	77.9	76.8	NA	NA
Stauffer et al ^37^ 2017						
LPD (n = 58)	25 (3‐100)	27 (9‐70)	84.5	75.9	66.5	43.3
OPD (n = 193)	35 (3‐140)	17 (1‐63)	79.8	73.5	67.5	24.3
Conrad et al ^38^ 2017						
LPD (n = 40)	25 (3‐80)	18 (6‐53)	87.5	61.1	82.5	50
OPD (n = 25)	30 (13‐60)	17 (9‐62)	84	45	76	44
Kuesters et al^39^ 2018						
LPD (n = 62)	28 (1‐75)	17 (7‐28)	87.0	NA	NA	20 (5‐y OS)
OPD (n = 278)	27 (1‐75)	16 (2‐47)	71.0	NA	NA	14 (5‐y OS)
Chen et al^40^ 2015						
RPD (n = 19)	30 ± 9	18.1 ± 6.6	94.7	NA	NA	23.0 mo (MST)
OPD (n = 38)	31 ± 10	17.8 ± 7.1	92.1	NA	NA	22.0 mo (MST)
Marino et al^41^ 2019						
RPD (n = 16)	NA	26 (19‐39)	93.7	87.5	85.7	65.2
OPD (n = 13)	NA	21 (14‐33)	76.9	84.6	80.0	62.
Kwon et al^42^ 2020						
Propensity score matched cohort (MIPD includes RPD and LPD)						
MIPD (n = 73)	27.5 (mean)	18.9 (mean)	76.7	80.8	84.9	44.7 (5‐y OS)
OPD (n = 219)	28.4 (mean)	21.3 (mean)	74.9	59.8	79.4	26.7 (5‐y OS)
Shi et al^43^ 2020						
RPD (n = 149)	NA	NA	NA	NA	28.4 mo (MST)	25.9 (5‐y OS)

Abbreviations: 1‐y OS, 1‐year survival rate; 3‐y OS, 3‐year survival rate; 5‐y OS, 5‐year survival rate; IQR, interquartile range; LN, lymph node; mo, months; MST, median survival time; NA, not available; R0, margin negative resection.

^a^ Median (IQR) was used.

^b^ IQR range.

The recently developed robotic surgical system has overcome the limitations of laparoscopic technology by providing an isometric 3D view and a high level of flexibility for manipulation. Robotic approaches for MIDP have been increasingly applied throughout the world. In Japan, robotic application for MIDP is covered by health insurance from April 2020. Although studies addressing the robotic benefits of MIDP are still few, it has been reported that robotic DP is as feasible and as safe as the laparoscopic and the conventional open approaches.^21^, ^22^ Daouadi et al^21^ evaluated the clinical data of 124 patients who underwent RDP (n = 30) or LPD (n = 94) between 2004 and 2011, and reported that RDP had a lower conversion rate, less blood loss, and shorter operating time compared to LDP. Chen et al^22^ evaluated the surgical outcomes in 80 MIDPs scheduled for spleen preservation (47 RDP and 33 LDP) and 39 MIDPs with splenectomy (22 RDP and 17 LDP). They reported that RDP was beneficial for the spleen‐preserving patients in the following aspects: less blood loss, less transfusion frequency, shorter operative time, overall spleen preserving rates, and shorter postoperative hospital stay compared to LDP. On the other hand, in patients who scheduled MIDP with splenectomy for malignant tumors, RDP had no advantages over LDP including oncologic outcomes such as lymph node retrieval, R0 resection rate, and use of adjuvant chemotherapy.^22^ Lai and Tang^23^ reported that RDP required a longer operative time than LDP, but there were no marked differences in blood loss, spleen‐preservation rate, postoperative hospital stay, or overall morbidity rate between the two groups. In a comparison of surgical outcomes among three types of DPs (RDP in 21, LDP in 25, and ODP in 43 cases) for benign and malignant diseases, operative time was longest in RDP (ODP < LDP < RDP) and blood loss was lowest in RDP (RDP < LDP < ODP).^24^ The rate of patients with Clavien‐Dindo ≥ grade III was lowest in RDP (RDP < LDP < ODP) and length of hospital stay was also shortest in RDP (RDP < LDP < ODP). Thus, RDP can provide less invasiveness in certain aspects.

## 3 MINIMALLY INVASIVE PANCREATODUODENECTOMY (MIPD)

Minimally invasive pancreatoduodenectomy (MIPD) is a challenging surgery because of its technical difficulty. The greatest difference between MIDP and MIPD is the presence in MIPD of anastomotic reconstruction such as cholangiojejunostomy, pancreatojejunostomy, and gastrojejunostomy. The laparoscopic procedure is unsuitable for the reconstruction process of LPD because of the difficulty in adjusting the axis of the forceps to adequate suturing lines of pancreatic or biliary reconstruction.^10^ Immature reconstruction substantially leads to postoperative complication such as clinically relevant pancreatic fistula/anastomotic leakage, resulting in prolonged hospital stay and even surgery‐related mortality. These situations also apply to OPD. According to the Miami international evidence‐based guidelines, insufficient data exist to recommend MIPD over OPD.^4^ LPD has been associated with less delayed gastric emptying, decreased blood loss and shorter hospital stay compared to OPD, without increasing overall costs.^7^, ^25^, ^26^ Lai and Tang^23^ comprehensively reviewed the LPDs and reported equivalent outcomes with respect to perioperative morbidity and mortality rates compared to OPDs, although the laparoscopic approach tended to have a longer operation time and less blood loss. These results may be difficult to generalize to other centers, because all these procedures were performed by experienced laparoscopic surgeons. Lai and Tang^23^ concluded that LPD was feasible and safe in well‐selected patients in experienced hands.

Three RCTs comparing LPD and OPD have been published.^27^, ^28^, ^29^ Two single‐center RCTs reported a shorter hospital stay in LPD.^28^, ^29^ On the other hand, a recent report from the Dutch Pancreatic Cancer Group (DPCG) has shown a risk of severe complications including postoperative death especially during the introduction of LPD compared to OPD.^27^ This report astonished pancreatic surgeons. The protocol of the LEOPARD 2 study (NTR5689) was published as a multicenter, patient‐blinded, randomized controlled phase 2/3 trial.^27^ Safety outcomes focused on the first part (phase 2 trial) including the initial 40 patients (20 LPDs and 20 OPDs). The safety monitoring board then assessed whether it would be safe to proceed to a phase 3 trial comparing time to functional recovery as a primary endpoint. Although the trial proceeded to phase 3, the safety monitoring board advised its early termination after randomization of 105 patients, of whom 99 underwent surgery (50 LPDs and 49 OPDs) (73% of the planned sample size), because of a high 90‐day complication‐related mortality of 10% in patients with LPD compared to 2% in OPD. The trial was very well planned and conducted by the four participating hospitals. Patient volume had to be at least 20 PDs annually. Every surgeon had to complete dedicated training programs for LDP (LAELAPS‐1)^30^ and LPD (LAELAPS‐2).^31^ In addition, all participating surgeons had experience of 50 or more advanced laparoscopic gastrointestinal procedures and 50 or more PDs (either LPD or OPD), and had performed at least 20 LPDs before trial participation. Nevertheless, five patients died within 90 days postoperatively during 8 months, although the mortality rates in the two groups (LPD and OPD) were not statistically different (*P* = .20). Finally, this multicenter RCT was stopped prematurely because of safety concerns as a result of higher 90‐day mortality in the LPD group.^27^ In this trial, the major complication (Clavien‐Dindo ≥ grade III) rate was comparable between LPD and OPD. Many studies suggest that MIPD should be limited to experienced surgeons in high‐volume centers because of the long learning curve and the difficulty of the procedure. LPD requires advanced skills in both pancreatic and laparoscopic surgery and there have been concerns as to the safety of its implementation. Thus, this approach has been safe and feasible when performed by experienced surgeons in high‐volume centers. Currently, no clear advantages of LPD compared with OPD have been demonstrated by RCTs, and LPD is still hard to generalize to untrained surgeons.

From pooled data in observational studies,^32^, ^33^, ^34^, ^35^, ^36^, ^37^, ^38^, ^39^, ^40^, ^41^, ^42^, ^43^ MIPD for PDAC provides comparable oncologic outcomes to OPD, as in MIDP (Table 1). Both MIPD and OPD are valid approaches for selected patients with PDAC. However, there are no comparative data about the indication for PDAC after neoadjuvant chemotherapy or requiring vascular resection. Robotic systems address many obstacles in LPD and may permit complex surgery to be performed with the same techniques as open surgery. Several steps of LPD may be improved by a robotic approach, including dissection of the pancreas from major vasculatures, lymph node dissection, dissection and resection of the uncinate process, and reconstruction of anastomoses. Several meta‐analyses have compared surgical outcomes between RPD and OPD.^44^, ^45^, ^46^ In these meta‐analyses, RPD had a significantly longer operation time and less blood loss compared to OPD, whereas pancreatic fistula and mortality were not significantly different between the two groups.^44^, ^45^, ^46^ The occurrence of clinically relevant pancreatic fistula (Grade B/C defined by the International Study Group of Pancreatic Fistula) in RPD was between 6.9% and 31.3%,^47^, ^48^, ^49^, ^50^, ^51^ and the superiority of RPD to OPD in clinically relevant pancreatic fistula is still controversial. Klompmaker et al^48^ have reported a significantly higher incidence of clinically relevant pancreatic fistula in MIPD (LPD and RPD) compared to OPD by a pan‐European propensity score‐matched analysis (23% and 13%, respectively). In a retrospective observational study using the NSQIP database, Zimmerman et al^52^ compared the short surgical outcomes among OPD (n = 6336, 92.8%), LPD (n = 280, 4.1%), and RPD (n = 211, 3.1%), and they concluded that LPD was independently associated with less morbidity after controlling for differences among the three groups. Studies of the oncologic efficacy of RPD have yielded equivalent results in terms of lymph node retrieval and positive margin status by meta‐analysis including various pancreatic pathologies.^44^, ^45^, ^46^ The oncologic benefit on PDAC derived from RPD remains uncertain, because of insufficient pooled data.^40^, ^41^, ^42^, ^43^ According to limited cohort studies,^40^, ^41^, ^42^, ^43^ the survival outcomes in PDAC are comparable between RPD and OPD (Table 1). In addition, there is no evidence of superiority between RPD and LPD. Although RPD may overcome some of the technical difficulties in LDP, its feasibility and safety need to be verified by the accumulation of clinical data.

## 4 LEARNING CURVE ANALYSIS AND HOSPITAL VOLUME

Education is one of the most important issues especially for MIPR, because pancreatic surgery is technically difficult even in an open operation, and there are few opportunities for training due to the rarity of the indicated lesions. In 2014, the DPCG initiated the nationwide Longitudinal Assessment and Realization of Laparoscopic Pancreatic Surgery (LAELAPS) program to safely implement minimally invasive pancreatic surgery.^30^ The LAELAPS‐1 program (LDP training) resulted in a seven‐fold increase in the use of LDP in the Netherlands and was followed by decreased conversion rates (from 38% to 8%) and hospital stay (from 9 to 7 days).^30^ Thereafter, the DPCG initiated the multicenter LAELAPS‐2 program (LPD training),^31^ which aimed to safely introduce LPD in a multicenter setting and to prospectively assess surgical outcomes. The DPCG have started the LAELAPS‐3 program on RPD. The standardization of operative procedures of MIPR contributes to shorter operative times and may enable pancreatic surgeons to bring out their full potential during the operations.

Depending on the surgical outcomes (mainly in the operative time and blood loss) that were used to assess the learning curve, 10‐20 cases have been proposed to be required to reach proficiency in LDP^53^, ^54^, ^55^, ^56^, ^57^, ^58^, ^59^, ^60^ (Table 2). For LPD, the surgeons in the three RCTs were required to have had experience of LPD, such as 25 LPD ≥ in the PLOT trial, 20 LPD ≥ in the PADULAP trial, and 20 LPD ≥ in the LEOPARD trial, prior to participation in the trials.^27^, ^28^, ^29^ In the LPD learning curve, related improvement in surgical outcomes of the operative time and blood loss was seen after 30‐50 cases^53^, ^61^, ^62^, ^63^, ^64^, ^65^ (Table 2). For robot‐assisted pancreatic resections (RDP and RPD), 20‐40 cases have been proposed as being needed to overcome the learning curve as an initial progress^40^, ^43^, ^66^, ^67^, ^68^, ^69^ (Table 2). From these learning curve analyses, an experienced surgeon may be defined as a surgeon who has experienced 20 cases in LDP and 30 cases in LPD. On the other hand, among 41 videos of LPD procedures in the LEOPARD‐2 trial, 22% received a technical summary score below average even after every surgeon completed a training program for LPD. Van Hilst et al^27^ discussed differences between the LEOPARD‐2 trial and the PLOT and PADULAP trials.^28^, ^29^ PLOT and PADULAP were single‐center trials, whereas four centers participated in the LEOPARD‐2 trial. The center in the PLOT trail had previously done over 150 LPDs, and all the procedures were performed by either of the two senior surgeons, who had sufficient experience of OPD and LPD (over 25 OPDs and 25 LPDs).^29^ In the PADULAP trial, the single expert surgeon, who had done 20 LPDs before the start of the trial, performed LPDs, but also had extensive experience in laparoscopic gastric bypass surgery with hand‐sewn anastomosis (more than 250 procedures).^28^ The necessity for comprehensive and continuous surgical training to achieve patient safety is clear. In Japan, the endoscopic surgical skill qualification system (ESSQS) was established by the Japan Society for Endoscopic Surgery to maintain and improve the quality of laparoscopic surgery. To become a qualified surgeon as judged by the ESSQS, applicants should have performed 20 advanced laparoscopic gastrointestinal surgeries (or 50 simple laparoscopic gastrointestinal surgeries such as cholecystectomy) as the chief surgeon. Additionally, they must submit a list of patients on whom they have performed surgery (including complications) and an unedited video showing the relevant surgical procedure, which are assessed by two judges. Qualification or educational training systems such as the nationwide LAELAPS program and Japanese ESSQS are useful to maintain and improve the quality of surgical techniques and to standardize minimally invasive pancreatic surgery.

**TABLE 2:** Required cases to attain proficiency in minimally invasive pancreatic surgery

Operative time	Blood loss	Hospital stay	Complication	Pancreatic fistula	Grade B/C^a^
Laparoscopic distal pancreatectomy					
De Rooji et al^54^ (N = 111)	No curve	No curve	30	30	30
Malleo et al^55^ (N = 100)	No curve	No curve	No curve	No curve	No curve
Ricci et al^56^ (N = 32)	17	NA	No curve	No curve	No curve
Braga et al^57^ (N = 30)	10	No curve	No curve	No curve	No curve
Barrie et al^58^ (N = 25)	10	6	No curve	NA	NA
Laparoscopic pancreaticoduodenectomy					
Nagakawa et al^62^ (N = 150)	30	30	NA	No curve	No curve
Lu et al^63^ (N = 120)	30	30	No curve	No curve	No curve
Kim et al^64^ (N = 100)	34	NA	34	34	34
Wang et al^65^ (N = 57)	11	11	No curve	No curve	No curve
Speicher et al^66^ (N = 56)	10	50	NA	NA	NA
Robot‐assisted distal pancreatectomy					
Klompmaker et al^67^ (N = 90)	31	NA	NA	NA	NA
Shyr et al^68^ (N = 70)	37	37	37	No curve	37
Robot‐assisted pancreaticoduodenectomy					
Shi et al^43^ (N = 450)	100, 250	100, 250	100	No curve	100
Boone et al^69^ (N = 200)	80, 140	20	No curve	No curve	40
Shyr et al^68^ (N = 61)	20	20	No curve	No curve	No curve
Chen et al^40^ (N = 60)	40	40	NA	No curve	NA

Note

No curve means that there is no significant improvement (no learning curve).

Abbreviation: NA, not available.

^a^ According to International Study Group of Pancreatic Fistula.

Although robotic assistance during MIPD may contribute to overcoming several aspects of technical difficulty in LPD, RPD also requires enough time and experience to reach proficiency. There are still a limited number of learning curve analyses in RPD and RDP.^40^, ^43^, ^66^, ^67^, ^68^ From these observational studies, experience with at least 20‐40 robot‐assisted pancreatic surgeries is required for the initial advance in the techniques. In a learning curve analysis from 450 cases of RPD by three operators at the Shanghai Ruijin Hospital during 8 years, Shi et al^43^ commented that there was a steady improvement during phase I (cases 1‐100), a “plateau” in phase II (cases 100‐250), and a further improvement in phase III (after experience of 250 cases) in operative outcomes such as operative time and blood loss. Four of five cases with conversion to laparotomy were found in phase I (80%). Additionally, the incidence of pancreatic leakage in the last 350 cases (phase II and III) was reported to be significantly lower than that in the first 100 cases (phase I) (15.1% and 30.0%, respectively).^43^ Shi et al^43^ recommended that surgeons could perform more difficult cases and expand the indications for RPD after 250 experiences. Boone et al^68^ also reported two inflexion points in operative time at case 80 and case 140 from their experience with 200 RPDs. Thus, from these learning curve analyses, operative time and blood loss, especially, in RPDs could be improved again after a stable phase, but this was not the case for complications. Regarding the oncological outcome in RPD, Boone et al^68^ reported that the number of lymph node retrievals was significantly improved after 80 cases. Although the R0 resection rate also showed improvement with experience, it did not reach statistical significance during the 200 RPDs.^68^ Furthermore, Shi et al^43^ have reported that the number of lymph node retrievals was greatly improved after 100 cases during 450 RPDs. However, it is still unclear whether or not a learning curve in robot‐assisted pancreatic surgery contributes to survival outcomes. To attain the level of an expert surgeon including oncological aspects, the far advanced experience of more than 100 RPDs may be requested, beyond the initial progress in surgical technique acquired by performing 20‐40 RPDs.

Hospital procedural volume has been reported to be important to decrease postoperative complications.^70^, ^71^ Adam et al^70^ reported that increasing hospital procedural volume of LPD was associated with improved surgical outcomes in up to 22 cases per year. A decreased complication rate was seen in high‐volume centers performing >20 MIPD/year^70^ or >20 total PD/year.^71^ To minimize the morbidity and mortality in the early stage of the learning curve, a new complicated technique should be introduced in high‐volume centers in the first instance. National clinical databases (NCDs) for patients with cancer or patients who have had surgery have been established in the United States and in several nations in the European Union and East Asia. Also in Japan, a NCD was established in 2010 by the Japanese Society of Gastrointestinal Surgery, and data entry began in 2011. Detailed information including preoperative co‐morbidity, postoperative complications, and 30‐ and 90‐day mortality is required in the major surgeries including pancreatic resection. Furthermore, preoperative registration to NCD is essential for LPD and robot‐assisted pancreatic surgery to monitor their safety. Such databases aim to establish an understanding of the national standard of clinical care and the trends in clinical treatment. Furthermore, they can lead to continuous improvement of surgical techniques and quality of care in the country, and allow easy comparisons of surgical outcomes between countries throughout the world.

## 5 CONCLUSIONS

Minimally invasive pancreatic resection has spread internationally and has yielded less invasiveness in certain aspects. MIDP for benign and low‐grade malignant tumors has established a secure position over ODP in its reduced invasiveness, although the long‐term oncological outcome of MIDP for PDAC needs to be evaluated by well‐conducted RCTs. The feasibility and safety of MIPD is still controversial compared with OPD. MIPD should be limited to experienced surgeons in high‐volume centers because of the long learning curve and the difficulty of the procedure. The development of educational systems, the centralization of high‐volume centers, and nationwide registration systems may accelerate the safe spread of MIPR. To achieve a true MIPR, pancreatic surgeons are required to undergo comprehensive and continuous surgical training.

## DISCLOSURE

Conflict of Interest: The authors have no conflicts of interest to declare.

## ETHICAL APPROVAL

The authors are accountable for all aspects of the work in ensuring that questions related to the accuracy or integrity of any part of the work are appropriately investigated and resolved.

## References

[1] Gagner M , Pomp A . Laparoscopic pylorus‐preserving pancreatoduodenectomy. Surg Endosc. 1994;8(5):408–10. 7915434 10.1007/BF00642443

[2] Cuschieri A , Jakimowicz JJ , van Spreeuwel J . Laparoscopic distal 70% pancreatectomy and splenectomy for chronic pancreatitis. Ann Surg. 1996;223(3):280–5. 8604908 10.1097/00000658-199603000-00008 PMC1235116

[3] Melvin WS , Needleman BJ , Krause KR , et al. Computer‐enhanced robotic telesurgery. Initial experience in foregut surgery. Surg Endosc. 2002;16(12):1790–2. 12239646 10.1007/s00464-001-8192-9

[4] Asbun HJ , Moekotte AL , Vissers FL , et al. The miami international evidence‐based guidelines on minimally invasive pancreas resection. Ann Surg. 2020;271(1):1–14. 31567509 10.1097/SLA.0000000000003590

[5] de Rooij T , Besselink MG , Shamali A , et al. Pan‐European survey on the implementation of minimally invasive pancreatic surgery with emphasis on cancer. HPB. 2016;18(2):170–6. 26902136 10.1016/j.hpb.2015.08.005 PMC4814598

[6] van Hilst J , de Rooij T , Abu Hilal M , et al. Worldwide survey on opinions and use of minimally invasive pancreatic resection. HPB. 2017;19(3):190–204. 28215904 10.1016/j.hpb.2017.01.011

[7] de Rooij T , Klompmaker S , Abu Hilal M , Kendrick ML , Busch OR , Besselink MG . Laparoscopic pancreatic surgery for benign and malignant disease. Nat Rev Gastroenterol Hepatol. 2016;13(4):227–38. 26882881 10.1038/nrgastro.2016.17

[8] Venkat R , Edil BH , Schulick RD , Lidor AO , Makary MA , Wolfgang CL . Laparoscopic distal pancreatectomy is associated with significantly less overall morbidity compared to the open technique: a systematic review and meta‐analysis. Ann Surg. 2012;255(6):1048–59. 22511003 10.1097/SLA.0b013e318251ee09

[9] Konstantinidis IT , Lewis A , Lee B , et al. Minimally invasive distal pancreatectomy: greatest benefit for the frail. Surg Endosc. 2017;31(12):5234–40. 28493165 10.1007/s00464-017-5593-y PMC5980234

[10] Ohtsuka T , Nagakawa Y , Toyama H , et al. A multicenter prospective registration study on laparoscopic pancreatectomy in Japan: report on the assessment of 1,429 patients. J Hepatobiliary Pancreat Sci. 2020;27(2):47–55. 31665567 10.1002/jhbp.695

[11] de Rooij T , van Hilst J , van Santvoort H , et al. Minimally invasive versus open distal pancreatectomy (LEOPARD): a multicenter patient‐blinded randomized controlled trial. Ann Surg. 2019;269(1):2–9. 30080726 10.1097/SLA.0000000000002979

[12] van Hilst J , Strating EA , de Rooij T , et al. Costs and quality of life in a randomized trial comparing minimally invasive and open distal pancreatectomy (LEOPARD trial). Br J Surg. 2019;106(7):910–21. 31012498 10.1002/bjs.11147 PMC6594097

[13] van Hilst J , de Rooij T , Klompmaker S , et al. Minimally invasive versus open distal pancreatectomy for ductal adenocarcinoma (DIPLOMA): a Pan‐European Propensity Score Matched Study. Ann Surg. 2019;269(1):10–7. 29099399 10.1097/SLA.0000000000002561

[14] van Hilst J , Korrel M , de Rooij T , et al. Oncologic outcomes of minimally invasive versus open distal pancreatectomy for pancreatic ductal adenocarcinoma: a systematic review and meta‐analysis. Eur J Surg Oncol. 2019;45(5):719–27. 30579652 10.1016/j.ejso.2018.12.003

[15] Yamamoto M , Zaima M , Yamamoto H , et al. New laparoscopic procedure for left‐sided pancreatic cancer‐artery‐first approach laparoscopic RAMPS using 3D technique. World J Surg Oncol. 2017;15(1):213. 29197396 10.1186/s12957-017-1284-3 PMC5712113

[16] Sahakyan MA , Kim SC , Kleive D , et al. Laparoscopic distal pancreatectomy for pancreatic ductal adenocarcinoma: Long‐term oncologic outcomes after standard resection. Surgery. 2017;162(4):802–11. 28756944 10.1016/j.surg.2017.06.009

[17] Buanes T , Edwin B . Long term oncological outcome of laparoscopic techniques in pancreatic cancer. World J Gastrointest Endosc. 2018;10(12):383–91. 30631402 10.4253/wjge.v10.i12.383 PMC6323502

[18] Shin SH , Kim SC , Song KB , et al. A comparative study of laparoscopic vs. open distal pancreatectomy for left‐sided ductal adenocarcinoma: a propensity score‐matched analysis. J Am Coll Surg. 2015;220(2):177–85. 25529901 10.1016/j.jamcollsurg.2014.10.014

[19] Stauffer JA , Coppola A , Mody K , Asbun HJ . Laparoscopic versus open distal pancreatectomy for pancreatic adenocarcinoma. World J Surg. 2016;40(6):1477–84. 26847665 10.1007/s00268-016-3412-6

[20] Raoof M , Nota C , Melstrom LG , et al. Oncologic outcomes after robot‐assisted versus laparoscopic distal pancreatectomy: analysis of the national cancer database. J Surg Oncol. 2018;118(4):651–6. 30114321 10.1002/jso.25170 PMC6386178

[21] Daouadi M , Zureikat AH , Zenati MS , et al. Robot‐assisted minimally invasive distal pancreatectomy is superior to the laparoscopic technique. Ann Surg. 2013;257(1):128–32. 22868357 10.1097/SLA.0b013e31825fff08

[22] Chen S , Zhan Q , Chen JZ , et al. Robotic approach improves spleen‐preserving rate and shortens postoperative hospital stay of laparoscopic distal pancreatectomy: a matched cohort study. Surg Endosc. 2015;29(12):3507–18. 25791063 10.1007/s00464-015-4101-5

[23] Lai EC , Tang CN . Current status of robot‐assisted laparoscopic pancreaticoduodenectomy and distal pancreatectomy: a comprehensive review. Asian J Endosc Surg. 2013;6(3):158–64. 23710970 10.1111/ases.12040

[24] Rodriguez M , Memeo R , Leon P , et al. Which method of distal pancreatectomy is cost‐effective among open, laparoscopic, or robotic surgery? Hepatobiliary Surg Nutr. 2018;7(5):345–52. 30498710 10.21037/hbsn.2018.09.03 PMC6230836

[25] Conlon KC , de Rooij T , van Hilst J , et al. Minimally invasive pancreatic resections: cost and value perspectives. HPB. 2017;19(3):225–33. 28268161 10.1016/j.hpb.2017.01.019

[26] de Rooij T , Lu MZ , Steen MW , et al. Minimally invasive versus open pancreatoduodenectomy: systematic review and meta‐analysis of comparative cohort and registry studies. Ann Surg. 2016;264(2):257–67. 26863398 10.1097/SLA.0000000000001660

[27] van Hilst J , de Rooij T , Bosscha K , et al. Laparoscopic versus open pancreatoduodenectomy for pancreatic or periampullary tumours (LEOPARD‐2): a multicentre, patient‐blinded, randomised controlled phase 2/3 trial. Lancet Gastroenterol Hepatol. 2019;4(3):199–207. 30685489 10.1016/S2468-1253(19)30004-4

[28] Poves I , Burdio F , Morato O , et al. Comparison of perioperative outcomes between laparoscopic and open approach for pancreatoduodenectomy: the PADULAP randomized controlled trial. Ann Surg. 2018;268(5):731–9. 30138162 10.1097/SLA.0000000000002893

[29] Palanivelu C , Senthilnathan P , Sabnis SC , et al. Randomized clinical trial of laparoscopic versus open pancreatoduodenectomy for periampullary tumours. Br J Surg. 2017;104(11):1443–50. 28895142 10.1002/bjs.10662

[30] de Rooij T , van Hilst J , Boerma D , et al. Impact of a nationwide training program in minimally invasive distal pancreatectomy (LAELAPS). Ann Surg. 2016;264(5):754–62. 27741008 10.1097/SLA.0000000000001888

[31] de Rooij T , van Hilst J , Topal B , et al. Outcomes of a multicenter training program in laparoscopic pancreatoduodenectomy (LAELAPS‐2). Ann Surg. 2019;269(2):344–50. 29099400 10.1097/SLA.0000000000002563

[32] Croome KP , Farnell MB , Que FG , et al. Total laparoscopic pancreaticoduodenectomy for pancreatic ductal adenocarcinoma: oncologic advantages over open approaches? Ann Surg. 2014;260(4):633–8; discussion 8–40. 25203880 10.1097/SLA.0000000000000937

[33] Dokmak S , Fteriche FS , Aussilhou B , et al. Laparoscopic pancreaticoduodenectomy should not be routine for resection of periampullary tumors. J Am Coll Surg. 2015;220(5):831–8. 25840531 10.1016/j.jamcollsurg.2014.12.052

[34] Song KB , Kim SC , Hwang DW , et al. Matched case‐control analysis comparing laparoscopic and open pylorus‐preserving pancreaticoduodenectomy in patients with periampullary tumors. Ann Surg. 2015;262(1):146–55. 25563866 10.1097/SLA.0000000000001079

[35] Delitto D , Luckhurst CM , Black BS , et al. Oncologic and perioperative outcomes following selective application of laparoscopic pancreaticoduodenectomy for periampullary malignancies. J Gastrointest Surg. 2016;20(7):1343–9. 27142633 10.1007/s11605-016-3136-9 PMC6033586

[36] Nussbaum DP , Adam MA , Youngwirth LM , et al. Minimally invasive pancreaticoduodenectomy does not improve use or time to initiation of adjuvant chemotherapy for patients with pancreatic adenocarcinoma. Ann Surg Oncol. 2016;23(3):1026–33. 26542590 10.1245/s10434-015-4937-x

[37] Stauffer JA , Coppola A , Villacreses D , et al. Laparoscopic versus open pancreaticoduodenectomy for pancreatic adenocarcinoma: long‐term results at a single institution. Surg Endosc. 2017;31(5):2233–41. 27604369 10.1007/s00464-016-5222-1

[38] Conrad C , Basso V , Passot G , et al. Comparable long‐term oncologic outcomes of laparoscopic versus open pancreaticoduodenectomy for adenocarcinoma: a propensity score weighting analysis. Surg Endosc. 2017;31(10):3970–8. 28205031 10.1007/s00464-017-5430-3

[39] Kuesters S , Chikhladze S , Makowiec F , et al. Oncological outcome of laparoscopically assisted pancreatoduodenectomy for ductal adenocarcinoma in a retrospective cohort study. Int J Surg. 2018;55:162–6. 29807171 10.1016/j.ijsu.2018.05.026

[40] Chen S , Chen JZ , Zhan Q , et al. Robot‐assisted laparoscopic versus open pancreaticoduodenectomy: a prospective, matched, mid‐term follow‐up study. Surg Endosc. 2015;29(12):3698–711. 25761559 10.1007/s00464-015-4140-y

[41] Marino MV , Podda M , Gomez Ruiz M , Fernandez CC , Guarrasi D , Gomez FM . Robotic‐assisted versus open pancreaticoduodenectomy: the results of a case‐matched comparison. J Robot Surg. 2019;14:493–502. 31473878 10.1007/s11701-019-01018-w

[42] Kwon J , Song KB , Park SY , et al. Comparison of minimally invasive versus open pancreatoduodenectomy for pancreatic ductal adenocarcinoma. A propensity score matching analysis. Cancers. 2020;12(4):982. 10.3390/cancers12040982 PMC7226374 32326595

[43] Shi Y , Wang W , Qiu W , et al. Learning curve from 450 cases of robot‐assisted pancreaticoduocectomy in a high‐volume pancreatic center: optimization of operative procedure and a retrospective study. Ann Surg. 2019 10.1097/SLA.0000000000003664. 31651533

[44] Yan Q , Xu LB , Ren ZF , Liu C . Robotic versus open pancreaticoduodenectomy: a meta‐analysis of short‐term outcomes. Surg Endosc. 2020;34(2):501–9. 31848756 10.1007/s00464-019-07084-3

[45] Peng L , Lin S , Li Y , Xiao W . Systematic review and meta‐analysis of robotic versus open pancreaticoduodenectomy. Surg Endosc. 2017;31(8):3085–97. 27928665 10.1007/s00464-016-5371-2

[46] Podda M , Gerardi C , Di Saverio S , et al. Robotic‐assisted versus open pancreaticoduodenectomy for patients with benign and malignant periampullary disease: a systematic review and meta‐analysis of short‐term outcomes. Surg Endosc. 2020;34(6):2390–409. 32072286 10.1007/s00464-020-07460-4

[47] Kim HS , Kim H , Kwon W , et al. Perioperative and oncologic outcome of robot‐assisted minimally invasive (hybrid laparoscopic and robotic) pancreatoduodenectomy: based on pancreatic fistula risk score and cancer/staging matched comparison with open pancreatoduodenectomy. Surg Endosc. 2020 10.1007/s00464-020-07551-2. 32277354

[48] Klompmaker S , van Hilst J , Wellner UF , et al. Outcomes after minimally‐invasive versus open pancreatoduodenectomy: a pan‐european propensity score matched study. Ann Surg. 2020;271(2):356–63. 29864089 10.1097/SLA.0000000000002850

[49] Zureikat AH , Postlewait LM , Liu Y , et al. A multi‐institutional comparison of perioperative outcomes of robotic and open pancreaticoduodenectomy. Ann Surg. 2016;264(4):640–9. 27433907 10.1097/SLA.0000000000001869

[50] Kauffmann EF , Napoli N , Menonna F , et al. A propensity score‐matched analysis of robotic versus open pancreatoduodenectomy for pancreatic cancer based on margin status. Surg Endosc. 2019;33(1):234–42. 29943061 10.1007/s00464-018-6301-2

[51] Giulianotti PC , Sbrana F , Bianco FM , et al. Robot‐assisted laparoscopic pancreatic surgery: single‐surgeon experience. Surg Endosc. 2010;24(7):1646–57. 20063016 10.1007/s00464-009-0825-4

[52] Zimmerman AM , Roye DG , Charpentier KP . A comparison of outcomes between open, laparoscopic and robotic pancreaticoduodenectomy. HPB. 2018;20(4):364–9. 29183703 10.1016/j.hpb.2017.10.008

[53] Dokmak S , Fteriche FS , Aussilhou B , et al. The largest European single‐center experience: 300 laparoscopic pancreatic resections. J Am Coll Surg. 2017;225(2):226–34.e2. 28414116 10.1016/j.jamcollsurg.2017.04.004

[54] de Rooij T , Cipriani F , Rawashdeh M , et al. Single‐surgeon learning curve in 111 laparoscopic distal pancreatectomies: does operative time tell the whole story? J Am Coll Surg. 2017;224(5):826–32.e1. 28126547 10.1016/j.jamcollsurg.2017.01.023

[55] Malleo G , Damoli I , Marchegiani G , et al. Laparoscopic distal pancreatectomy: analysis of trends in surgical techniques, patient selection, and outcomes. Surg Endosc. 2015;29(7):1952–62. 25303912 10.1007/s00464-014-3890-2

[56] Ricci C , Casadei R , Buscemi S , et al. Laparoscopic distal pancreatectomy: what factors are related to the learning curve? Surg Today. 2015;45(1):50–6. 24610347 10.1007/s00595-014-0872-x

[57] Braga M , Ridolfi C , Balzano G , Castoldi R , Pecorelli N , Di Carlo V . Learning curve for laparoscopic distal pancreatectomy in a high‐volume hospital. Updates Surg. 2012;64(3):179–83. 22763577 10.1007/s13304-012-0163-2

[58] Barrie J , Ammori BJ . Minimally invasive distal pancreatectomy: a single‐center analysis of outcome with experience and systematic review of the literature. Surg Laparosc Endosc Percutan Tech. 2015;25(4):297–302. 26147049 10.1097/SLE.0000000000000185

[59] Benizri EI , Germain A , Ayav A , et al. Short‐term perioperative outcomes after robot‐assisted and laparoscopic distal pancreatectomy. J Robot Surg. 2014;8(2):125–32. 27637522 10.1007/s11701-013-0438-8

[60] Kneuertz PJ , Patel SH , Chu CK , et al. Laparoscopic distal pancreatectomy: trends and lessons learned through an 11‐year experience. J Am Coll Surg. 2012;215(2):167–76. 22632910 10.1016/j.jamcollsurg.2012.03.023

[61] Nagakawa Y , Nakamura Y , Honda G , et al. Learning curve and surgical factors influencing the surgical outcomes during the initial experience with laparoscopic pancreaticoduodenectomy. J Hepatobiliary Pancreat Sci. 2018;25(11):498–507. 30291768 10.1002/jhbp.586

[62] Lu C , Jin W , Mou YP , et al. Analysis of learning curve for laparoscopic pancreaticoduodenectomy. J Vis Surg. 2016;2:145. 29078532 10.21037/jovs.2016.07.25 PMC5637755

[63] Kim SC , Song KB , Jung YS , et al. Short‐term clinical outcomes for 100 consecutive cases of laparoscopic pylorus‐preserving pancreatoduodenectomy: improvement with surgical experience. Surg Endosc. 2013;27(1):95–103. 22752284 10.1007/s00464-012-2427-9

[64] Wang M , Meng L , Cai Y , et al. Learning curve for laparoscopic pancreaticoduodenectomy: a CUSUM analysis. J Gastrointest Surg. 2016;20(5):924–35. 26902090 10.1007/s11605-016-3105-3

[65] Speicher PJ , Nussbaum DP , White RR , et al. Defining the learning curve for team‐based laparoscopic pancreaticoduodenectomy. Ann Surg Oncol. 2014;21(12):4014–9. 24923222 10.1245/s10434-014-3839-7

[66] Klompmaker S , van der Vliet WJ , Thoolen SJ , et al. Procedure‐specific training for robot‐assisted distal pancreatectomy. Ann Surg. 2019 10.1097/SLA.0000000000003291 30946088

[67] Shyr BU , Chen SC , Shyr YM , Wang SE . Learning curves for robotic pancreatic surgery‐from distal pancreatectomy to pancreaticoduodenectomy. Medicine. 2018;97(45):e13000. 30407289 10.1097/MD.0000000000013000 PMC6250552

[68] Boone BA , Zenati M , Hogg ME , et al. Assessment of quality outcomes for robotic pancreaticoduodenectomy: identification of the learning curve. JAMA Surg. 2015;150(5):416–22. 25761143 10.1001/jamasurg.2015.17

[69] Takahashi C , Shridhar R , Huston J , Meredith K . Outcomes associated with robotic approach to pancreatic resections. J Gastrointest Oncol. 2018;9(5):936–41. 30505596 10.21037/jgo.2018.08.04 PMC6219977

[70] Adam MA , Thomas S , Youngwirth L , Pappas T , Roman SA , Sosa JA . Defining a hospital volume threshold for minimally invasive pancreaticoduodenectomy in the United States. JAMA Surg. 2017;152(4):336–42. 28030713 10.1001/jamasurg.2016.4753 PMC5470427

[71] Tran TB , Dua MM , Worhunsky DJ , Poultsides GA , Norton JA , Visser BC . The first decade of laparoscopic pancreaticoduodenectomy in the United States: costs and outcomes using the nationwide inpatient sample. Surg Endosc. 2016;30(5):1778–83. 26275542 10.1007/s00464-015-4444-y

